# Chebulinic Acid Suppresses Adipogenesis in 3T3-L1 Preadipocytes by Inhibiting PPP1CB Activity

**DOI:** 10.3390/ijms23020865

**Published:** 2022-01-13

**Authors:** Jinsoo Kim, Dohee Ahn, Sang J. Chung

**Affiliations:** 1 School of Pharmacy, Sungkyunkwan University, Suwon 16419, Korea; neto543@naver.com (J.K.); ehgml94@naver.com (D.A.); 2 Department of Biopharmaceutical Convergence, Sungkyunkwan University, Suwon 16419, Korea

**Keywords:** obesity, adipogenesis, 3T3-L1 adipocyte, chebulinic acid, natural product, PPP1CB

## Abstract

Depletion of protein phosphatase-1 catalytic subunit beta (PPP1CB), a serine/threonine protein phosphatase and potent adipogenic activator, suppresses the differentiation of 3T3-L1 preadipocytes into mature adipocytes. Therefore, PPP1CB is considered as a potential therapeutic target for obesity. We screened 1033 natural products for PPP1CB inhibitors and identified chebulinic acid, which is abundantly present in the seeds of *Euphoria longana* and fruits of *Terminalia chebula*. Chebulinic acid strongly inhibited the hydrolysis of 6,8-difluoro-4-methylumbelliferyl phosphate by PPP1CB (IC_50_ = 300 nM) and demonstrated potent antiadipogenic effects in 3T3-L1 preadipocytes in a concentration-dependent manner. Additional studies have demonstrated that chebulinic acid suppresses early differentiation by downregulating key transcription factors that control adipogenesis in 3T3-L1 cells. These results suggested that chebulinic acid may be a potential therapeutic agent for treating obesity by inhibiting PPP1CB activity.

## 1. Introduction

Obesity is a global health problem [1]. A World Health Organization report (2020) showed that 13% and 39% of adults aged 18 years and above are obese and overweight, respectively [2]. Obesity is characterized by an expansion in size and an increase in the number of adipocytes, caused by an imbalance between food intake and energy expenditure [3,4]. Numerous studies have shown that obesity leads to various complications, such as type 2 diabetes, atherosclerosis, high blood pressure, hyperlipidemia, non-alcoholic steatohepatitis, and cardiovascular diseases [5,6,7]. Because of the limits of lifestyle change and the surgical burdens of diseases, pharmacological strategies, such as the use of appetite suppressors, GLP-1 receptor agonists, or pancreatic lipase inhibitors, have been widely used to manage obesity [8,9]. However, since monotherapies showed limited effects by recruiting counter-regulatory pathways, the compounds targeting novel pathways are currently required as a concomitant drug with conventional drugs for obesity treatment [10]. Hence, natural products with higher safety and fewer side effects are currently developed as treatment for the obesity [11]. 

The most widely used cells for in vitro adipocyte research are 3T3-L1 preadipocytes [12]. In an appropriate medium composition, cells differentiate into adipocytes and accumulate lipid droplets [13]. The differentiation of 3T3-L1 preadipocytes was triggered by exposure to media supplemented with insulin, dexamethasone, isobutylmethylxanthine (IBMX), and fetal bovine serum (FBS), initiating a cascade of transcription factors, such as CCAAT/enhancer-binding protein (C/EBP)-1β and C/EBPδ, subsequently inducing the expression of peroxisome proliferator-activated receptor (PPAR)-γ and C/EBPα [14]. Differentiated 3T3-L1 adipocytes also secrete several adipokines, such as adiponectin and aP2 [15,16]. 

Protein phosphatase 1 (PPP1) is the most ubiquitously expressed type 1 serine/threonine phosphatase that regulates various metabolic functions [17]. PPP1 consists of a catalytic subunit and a regulatory subunit. In mammalian cells, three separate genes, protein phosphatase 1 catalytic subunit alpha (*PPP1CA*), protein phosphatase 1 catalytic subunit (*PPP1CB*), and protein tyrosine phosphatase 1 catalytic subunit gamma (*PPP1CC*), encode PPP1C, and PPP1C isoforms are alternatively spliced and transcribed [18]. A previous study by our team found that PPP1CB upregulated adipogenesis by modulating the early adipogenic process [19]. Considering all these facts, the identification of PPP1CB inhibitors is a good strategy to find potent therapeutic medicines for obesity.

Our team has been conducting research to discover natural products with inhibitory effects on protein phosphatases to ameliorate metabolic diseases, and to reveal the efficacy of natural products using cell-based assays. As part of our ongoing efforts to discover new anti-obesity natural products, we verified the inhibitory effects of 1033 natural products on PPP1CB using the 6,8-difluoro-4-methylumbelliferyl phosphate (DiFMUP) inhibition assay. Chebulinic acid was selected as a PPP1CB inhibitor (IC_50_ = 300 nM) in screening experiments and showed anti-adipogenic effects in 3T3-L1 adipocytes in a concentration-dependent manner. 

Chebulinic acid commonly exists in *Terminalia chebula* [20]. *T. chebula* shows pharmacological activities, such as antioxidant, anti-obesity, and anti-cancer effects [21]. While the anti-obesity effect of *T. chebula* extract has been published, chebulinic acid, one of compounds in *T. chebula* extract, has not been studied as an anti-obesity agent [22]. 

Chebulinic acid mostly affected the early stages of differentiation and downregulated key transcriptional factors that regulate adipogenesis in 3T3-L1 adipocytes. These results indicate that chebulinic acid is a potential therapeutic agent for obesity. 

## 2. Results

### 2.1. Purification of PPP1CB with Various Affinity Tags

To find the optimal affinity tag for the purification and enzymatic assays of PPP1CB, we utilized maltose-binding protein (MBP), glutathione-S-transferase (GST), and hexahistidine (6xHis) tags. Both GST- and MBP-tagged PPP1CBs were purified in a single step (Figure S2A,B). For determining kinetic constants, purified PPP1CB was added to the reaction buffer containing various concentrations of DiFMUP. The hydrolysis of DiFMUP to DiFMU was monitored on a plate reader at Ex/Em = 355/460 nm once every minute. The amount of product formation was calculated from the standard curve of fluorescence intensity over DiFMU concentration, which was obtained at the same buffer condition with the enzyme kinetics experiments (Figure S3). GST-tagged PPP1CB exhibited enzymatic activity, whereas MBP-tagged PPP1CB showed no enzymatic activity (Table 1). In the case of 6xHis-tagged PPP1CB, proteins purified using talon resins still had many impurities (Figure S2C-1). After cleavage of 6xHis tags using caspase-3, protein solutions were loaded in talon resins to eliminate caspase-3 and 6xHis tags (Figure S2C-2). PPP1CB was purified using a size-exclusion column, Superose-6. The final purified PPP1CB showed a more than 5-fold higher enzymatic activity than the 6xHis-tagged PPP1CB in terms of *k*_cat_/*K*_M_ (Table 1). De-tagged PPP1CB showed better enzymatic activity than GST-tagged PPP1CB (Table 1). Considering these findings, PPP1CB without an affinity tag was considered the most suitable for enzymatic assays. 

### 2.2. Screening of PPP1CB Inhibitors and Discovery of Novel Anti-Adipogenic Natural Products

**Figure 1:**
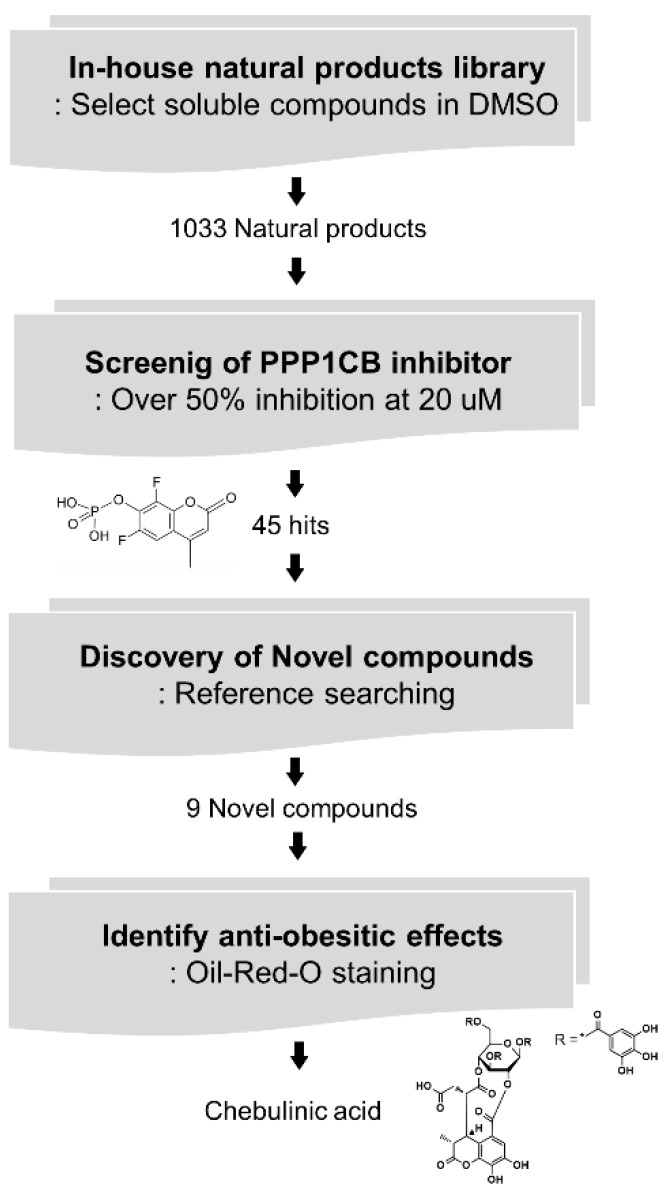
Identification of chebulinic acid as an anti-obesity drug candidate by screening of 1033 natural products for PPP1CB inhibitors. A total of 1033 natural products were transferred to black/black bottom 96-well plates and enzymatic screening was performed with PPP1CB. Inhibitory activity of each compound was evaluated using 6,8-difluoro-4-methylumbelliferyl phosphate (DiFMUP)-based assays, and the inhibitory effects of the 1033 natural products on PPP1CB were calculated using control-based normalization. The top 45 hits were selected. Among the 45 hits, nine compounds that have not reported anti-adipogenic effects were sorted based on literature search. These nine selected compounds were evaluated using oil red O staining to determine whether they suppress adipogenesis in 3T3-L1 cells. Finally, chebulinic acid was selected as the most potent compound for obesity treatment.

**Figure 2:**
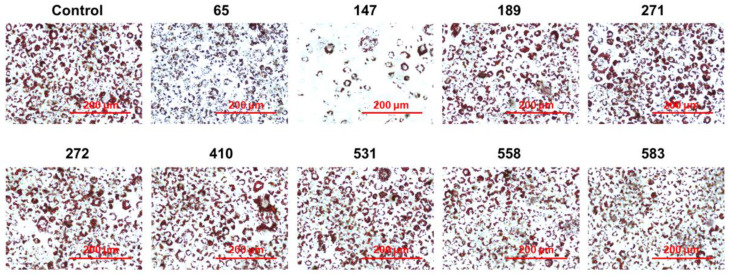
Anti-adipogenic activities of the nine selected compounds. After sorting out 45 hits by enzymatic assays, nine compounds were selected as novel compounds for the investigation of their anti-adipogenic activity. To validate the anti-adipogenic effects of the nine compounds, the cells were cultured until reaching 100% confluency. Then, the cells were treated with DMI containing 20 μL of indicated compound or 0.1% DMSO as control. After 6 days of differentiation, the cells were fixed with 4% paraformaldehyde. Then, the fixed cells were stained by oil red O solutions and washed with distilled water. The stained cells were captured by a Cytation 7 cell imaging multimode reader; 65: alpha-boswellic acid; 147: chebulinic acid; 189: cyanidin chloride; 271: gallic acid ethyl ester; 272: gallocatechin gallate; 410: medicagenic acid; 531: quercetin 7-rhamnoside; 558: salvianolic acid c; 583: sennoside B. Scale bar: 200 μm.

A commercial natural product library consisting of 1033 compounds isolated from traditional oriental medicinal plants were screened using a DiFMUP-based inhibition assay to identify strong inhibitors of PPP1CB. The catalytic activity of PPP1CB was assayed using the surrogate substrate, DiFMUP. Compounds (20 μM) were incubated with DiFMUP, and a final protein concentration of up to 1.5 nM was added to the buffer. Forty-eight hits showed PPP1CB inhibitory effects, out of which nine compounds had not yet been studied for their effects on adipogenesis in vitro and in vivo (Figure 1). To validate the anti-adipogenic effects of these nine compounds, we performed an oil red O staining assay and found that chebulinic acid exhibited anti-adipogenic effects in 3T3-L1 cells (Figure 2).

### 2.3. Inhibitory Effects and Cytotoxicity of Chebulinic Acid in PPP1CB and 3T3-L1 Cells

**Figure 3:**
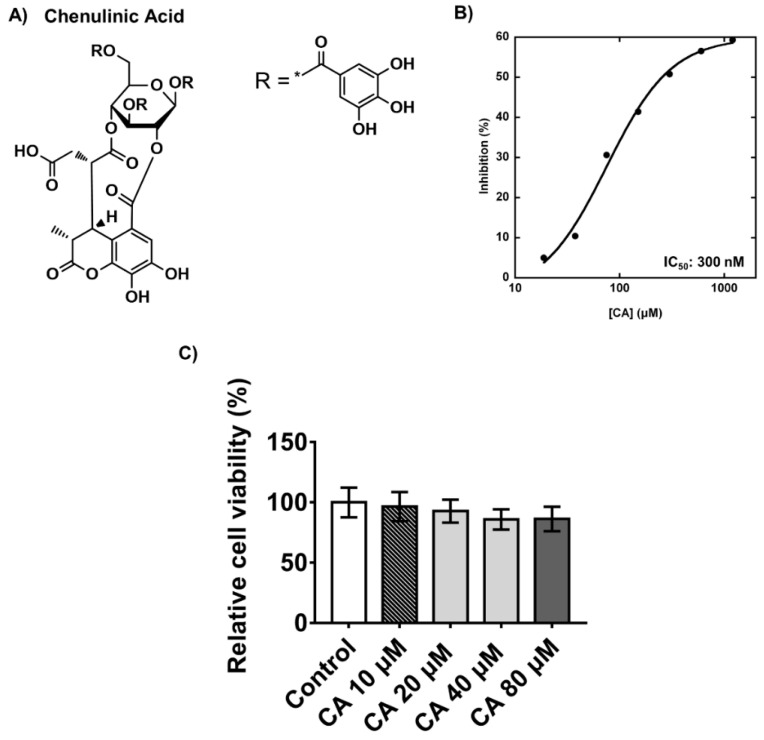
Inhibitory effect of chebulinic acid on PPP1CB. (**A**) Chemical structure of chebulinic acid (CA); (**B**) the half-maximal inhibitory concentration (IC_50_) of chebulinic acid against PPP1CB catalytic activity. Chebulinic acid was diluted from 12 to 0.019 μM in dimethyl sulfoxide (DMSO). Next, 10 μL of diluted chebulinic acid was added to 80 μL of reaction buffer containing 304 μM DiFMUP. Next, 10 μL PPP1CB (1.5 nM of final concentration) was added to the reaction buffer containing DiFMUP and chebulinic acid. IC_50_ value was calculated using the sigmoid dose–response model with the seven data points; (**C**) cytotoxicity of chebulinic acid on 3T3-L1 preadipocytes. Cell viabilities were measured using the water-soluble tetrazolium (WST)-1 assay. Results are presented as the mean ± standard deviation.

Since chebulinic acid was identified as a novel compound that suppressed adipogenesis, we measured the half-maximal inhibitory concentration (IC_50_) values of chebulinic acid on PPP1CB. Chebulinic acid in dimethyl sulfoxide (DMSO) was added to a reaction buffer containing 304 μM DiFMUP, which is twice the *K*_M_ value. Next, PPP1CB was added to the reaction buffer and the enzyme reaction was monitored, showing a fluorescence increase at Ex/Em = 355/460 nm on a plate reader. The IC_50_ value for the inhibition of PPP1CB was calculated using a sigmoid dose–response model using KaleidaGraph software. The IC_50_ value of chebulinic acid for PPP1CB inhibition was 300 nM (Figure 3B). Confluency of 3T3-L1 preadipocytes is an important factor in adipogenesis; therefore, it is very important to evaluate the cytotoxicity of compounds that suppress adipogenesis. To validate the cytotoxicity of chebulinic acid, 3T3-L1 preadipocytes were incubated with 10, 20, 40, or 80 μM of the compound for 48 h. Cytotoxicity was determined using a water-soluble tetrazolium (WST)-1 assay. Chebulinic acid had showed a very low cytotoxicity (92% cell viability) up to 20 μM and a low cytotoxicity (84% cell viability) in the range from 40 μM to 80 μM (Figure 3C).

### 2.4. Chebulinic Acid Suppressed Adipogenesis of 3T3-L1 Cells in Concentration-Dependent Manner

**Figure 4:**
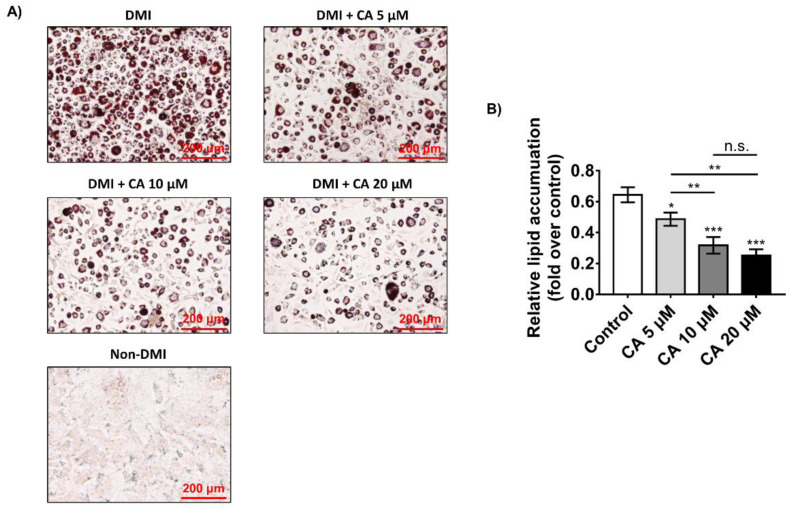
Effects of different concentrations of chebulinic acid (CA) on dexamethasone, methylisobutylxanthine, and insulin (DMI)-induced differentiation of 3T3-L1 preadipocytes. (**A**) Fully cultured 3T3-L1 preadipocytes were treated with DMI containing 5, 10, or 20 μM chebulinic acid or 0.1% DMSO as control group. After confirming fully differentiated cells in the control group, the cells were fixed with 4% paraformaldehyde and stained with 0.3% oil red O solution. After washing two times with distilled water, representative images of the cells were captured by a Cytation 7 cell imaging multimode reader. Scale bar: 200 μm; (**B**) quantification of the amount of fat. oil red O in adipocyte was extracted with isopropanol and transferred into the microwell plate followed by detection of absorption at 520 nm on a microplate reader. The results are presented as the mean ± standard deviation (SD, *n* = 3); * *p* < 0.05, ** *p* < 0.01, and *** *p* < 0.001 compared to the control group. Statistical analysis was performed by one-way ANOVA for multiple comparisons followed by Tukey’s test.

Next, we performed oil red O staining to elucidate the effects of chebulinic acid on the adipogenesis of 3T3-L1 preadipocytes into adipocytes. Dexamethasone, methylisobutylxanthine, and insulin (DMI) were added to 3T3-L1 adipocytes after reaching 100% confluency for 8 days. Since chebulinic acid has no cytotoxic effect at 20 μM, various concentrations of chebulinic acid (20, 10, and 5 μM) were used during differentiation. Next, the cells were fixed in 4% paraformaldehyde and stained with 0.3% oil red O solution. As shown in Figure 4A, the cells incubated in the DMI medium had a significantly increased number of lipid droplets compared with the cells incubated in the non-DMI medium. These results indicated that 3T3-L1 preadipocytes were successfully differentiated into adipocytes. As shown in the figure, chebulinic acid potently inhibited the production of lipid droplets in fully differentiated 3T3-L1 adipocytes. To quantify the stained cellular lipid amounts, the cells were incubated with isopropanol, and the oil red O solutions were analyzed at 520 nm using a microplate reader. Chebulinic acid potently suppressed adipogenesis, showing that chebulinic acid treatment reduced lipid accumulation in fully differentiated 3T3-L1 adipocytes in a concentration-dependent manner by 20–60% compared with the DMI treatment group (Figure 4B). Specifically, 20 μM chebulinic acid suppressed lipid accumulation by >60% suppression of lipid accumulations.

### 2.5. Chebulinic Acid Mainly Suppressed the Adipogenesis of 3T3-L1 Cells in the Early Stage

**Figure 5:**
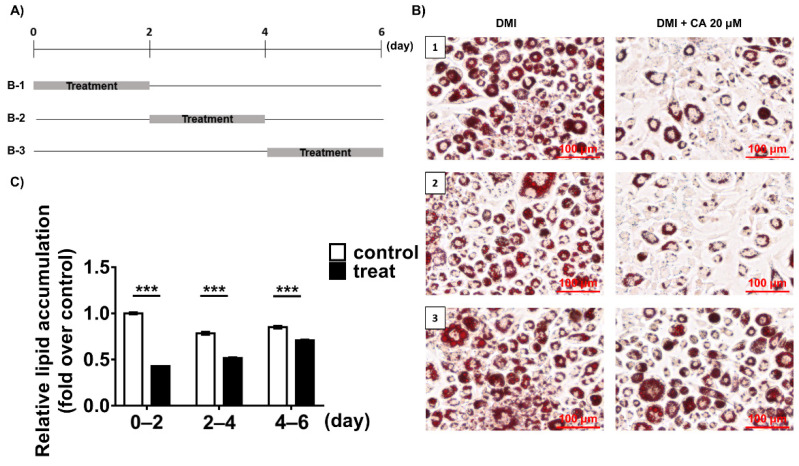
Effects of chebulinic acid (CA) on dexamethasone, methylisobutylxanthine, and insulin (DMI)-induced differentiation of 3T3-L1 preadipocytes in different stages of cells. (**A**) The schedule for chebulinic acid treatment during the differentiation of 3T3-L1 preadipocytes to adipocytes. Cells were treated with chebulinic acid (20 μM) on day 0–2 (B-1), day 2–4 (B-2), or day 4–6 (B-3) during differentiation by DMI treatment; (**B**) representative images of oil red O-stained cells. Each number on the picture corresponds to the number listed vertically (B-1, B-2, B-3) in Figure 5A. Scale bar: 100 μm; (**C**) after the extraction of oil red O from the stained cells, their absorbance was measured at 520 nm. The results are presented as the mean ± standard deviation (SD, *n* = 3); *** *p* < 0.001 compared to the control group.

Next, for deeper examination of the effects of Chebulinic acid on the 3T3-L1 adipogenesis, 20 μM of chebulinic acid was added into the cells in three different adipogenic stages, the early stage (days 0–2), the postmitotic intermediate stage (days 2–4), or the terminal stage (after days 4) (Figure 5A). After 6 days of differentiation of the 3T3-L1 preadipocytes, adipogenesis of the cells was visualized by using oil red O staining assays. Chebulinic acid significantly inhibited about 57% of the lipid accumulation in droplets upon its treatment on the early stages of adipogenesis (Figure 5B,C). On the other hand, treatment of chebulinic acid on the middle stage of adipogenesis presented a weaker anti-adipogenic effect than that on the early stage, showing a 38% inhibition of the lipid accumulation. In addition, treatment of chebulinic acid on the terminal stage exhibited a marginal effect compared to the non-treatment group (Figure 5B,C). These results indicated that chebulinic acid mainly affected the early stage of adipogenesis in 3T3-L1 cells in accordance with those in the knock-down of PPP1CB [19].

### 2.6. Chebulinic Acid Downregulated the Adipogenic Transcriptional Factors and Adipokines

**Figure 6:**
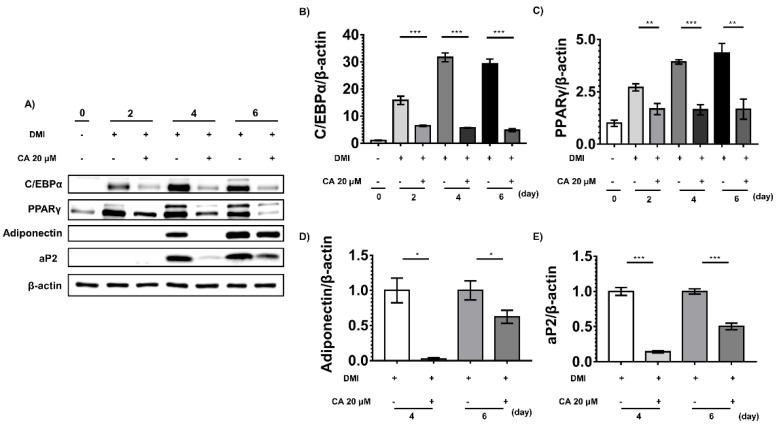
Effects of chebulinic acid (CA) on adipogenic factors and adipocyte markers. (**A**) Protein expression levels of adipogenic factors and adipocyte markers were detected three times independently using Western blotting. At the indicated time, 3T3-L1 cells were differentiated with dexamethasone, methylisobutylxanthine, and insulin (DMI) in the presence of 20 μM chebulinic acid or 0.1% dimethyl sulfoxide (DMSO) as a negative control; (**B**,**C**) the quantified graphs of C/EBPα (**B**) and PPARγ (**C**) normalized to non-differentiated group were shown; (**D**,**E**) since aP2 and adiponectin were not detected until day 2, these were normalized to control group in each treated day. Results are presented as the mean ± standard deviation (SD, *n* = 3) of three independent experiments; * *p* < 0.05, ** *p* < 0.01, and *** *p* < 0.001 compared with the control group.

To further study the anti-adipogenic effects of chebulinic acid in vitro, we conducted Western blotting to detect adipogenic factors and adipokines, including PPARγ, C/EBPα, adiponectin, and aP2. Before inducing adipogenesis, cells rarely express adipogenic factors, or adipokines. However, after inducing differentiation, the expression levels of PPARγ and C/EBPα were significantly increased on day 4. Adipokines, such as adiponectin and aP2, were highly expressed on day 6 (Figure 6). However, in the chebulinic acid treatment group, PPARγ and C/EBPα levels were significantly suppressed compared to those in the non-treatment group at all stages (Figure 6B,C). Chebulinic acid also potently suppressed the expression levels of the adipocyte markers adiponectin and aP2 after the cells induced the expression of adipokines (Figure 6D,E).

### 2.7. Chebulinic Acid Suppressed the mRNA Expression of Factors Associated with the Adipogenesis of 3T3-L1 Cells

**Figure 7:**
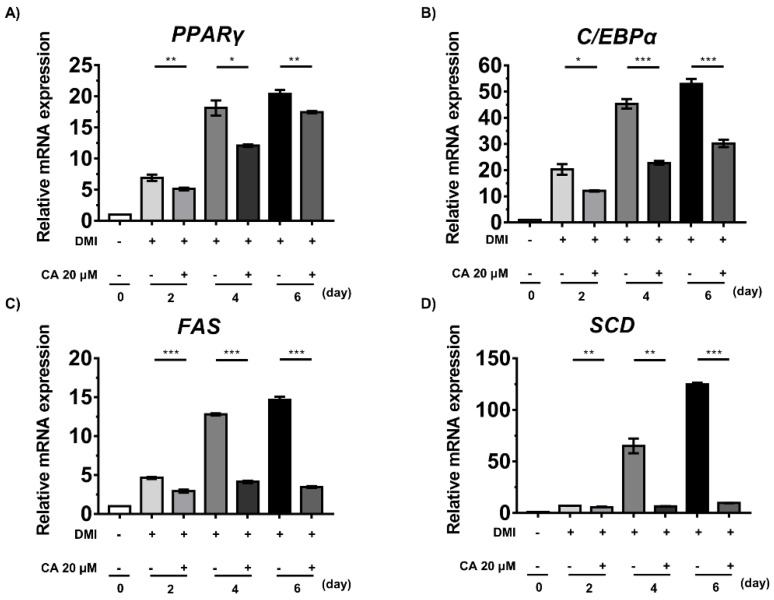
Effect of chebulinic acid (CA) on the mRNA expression levels of genes related to adipogenesis of 3T3-L1 cells induced by dexamethasone, methylisobutylxanthine, and insulin (DMI). The mRNA levels of *PPARγ* (**A**), *C/EBPα* (**B**), *FAS* (**C**), and *SCD* (**D**) were quantified using RT-PCR and normalized to those of *GAPDH* as an internal control. Results are presented as the mean ± standard deviation (SD, *n* = 3) of three independent experiments; * *p* < 0.05, ** *p* < 0.01, and *** *p* < 0.001 compared to the control group.

We further studied whether chebulinic acid regulated the expression levels of adipogenic genes using a reverse transcription–polymerase chain reaction (RT-PCR) to measure the mRNA levels of PPARγ, C/EBPα, fatty acid synthase (FAS), and stearoyl-CoA desaturase (SCD). In the control group, the expression levels of adipogenic genes were augmented by the induction of differentiation with DMI (Figure 7). In the chebulinic acid-treated group, PPARγ and C/EBPα expression levels were significantly decreased in the early stages, thereby decreasing the expression levels of FAS and SCD (Figure 7). In particular, FAS and SCD mRNA levels were considerably lower in the treatment group than in the non-treatment group from day 2 of differentiation. These results strongly suggest that chebulinic acid suppresses the adipogenesis and differentiation of 3T3-L1 preadipocytes into adipocytes at the transcriptional and translational levels (Figure 6 and Figure 7).

## 3. Discussion

Obesity poses several health risks worldwide [23]. It causes severe health problems along with other diseases, including diabetes, heart disease, respiratory problems, and osteoarthritis [24]. With a healthy diet and regular physical activity, pharmaceutical drugs are recommended to ameliorate obesity [25]. The treatment of obesity with chemical drugs commonly causes adverse effects and drug dependence [26]. For these reasons, researchers have been interested in identifying bioactive compounds with higher safety for the treatment of obesity [7]. Recently, natural substances have been shown to exert anti-obesity effects by modulating the digestive system, differentiation of adipocytes, thermogenic gene expression, lipolysis, and gut microbiota [7].

Adipocytes are the most important factors controlling energy metabolism in the body, and their malfunction can cause severe metabolic disorders that result in excessive energy storage in these cells, leading to obesity [27,28,29]. In addition to adipocyte dysfunction, overfeeding can cause an increase in the size of lipid droplets; this is called hypertrophy or induced adipogenesis, and it causes hyperplasia, which is an increase in the number of adipocytes [30,31]. Mouse embryo-derived 3T3-L1 cells are widely used to validate anti-obesity drugs in vitro [32]. 

C/EBPβ and C/EBPδ are induced by DMI, leading to PPARγ stimulation. Activated PPARγ induces C/EBPα, and these transcription factors regulate the expression of adipogenic genes, such as FABP4, ACC, FAS, and HSL [33,34]. The relationship between obesity and several types of protein phosphatases, including protein tyrosine phosphatase and serine/threonine phosphatase, has been recently studied [35]. In previous studies, our team demonstrated the adipogenic effect of PPP1CB, a catalytic subunit beta isoform of type 1 protein phosphatase 1. Our team also showed that the expression level of PPP1CB increased in the early stages and knockdown of the PPP1CB downregulated adipogenic gene and protein expression in the early stages, indicating that PPP1CB plays an important role in the early stages of adipogenesis [19].

In this study, 1033 compounds derived from traditional medical plants were screened to identify potent PPP1CB inhibitors. Among the 45 compounds that showed inhibition percentages of over 50% at 20 µM, 39 compounds had already been validated to show anti-obesity effects. Current results suggest that PPP1CB may be the target of these compounds for the anti-obesity effect. To identify novel compounds for obesity, the remaining nine compounds were investigated using cell-based assays to further study their anti-obesity effects using oil red O staining. Only chebulinic acid showed an inhibitory effect on 3T3-L1 cell adipogenesis. Chebulinic acid was not toxic to 3T3-L1 cells at a concentration of 20 µM but showed a strong inhibitory effect on adipogenesis in a concentration-dependent manner. Among the three treatment conditions, chebulinic acid showed the strongest inhibitory effect at day 0–2. These results indicate that chebulinic acid mainly suppresses adipogenesis in 3T3-L1 adipocytes, similar to the knockdown of PPP1CB.

In previous studies, chebulinic acid was shown to have an antagonistic effect on type 2 diabetes by inhibiting the protein tyrosine phosphatase non-receptor (PTPN)-11 and PTPN9 [36]. Thus, it is possible that the anti-obesity effects of chebulinic acid are caused by the inhibition of PTPN11 and PTPN9. However, based on the report that PTPN11 suppresses the adipogenesis of 3T3-L1 cells, we ruled out the possibility that the anti-obesity effects of chebulinic acid were due to PTPN11 inhibition [37]. PTPN9 has been identified as an anti-diabetic target but its relation to adipogenesis has not been reported [38]. These results indicate that the anti-adipogenic effects of chebulinic acid were mainly caused by the inhibition of PPP1CB. 

We further validated the efficacy of chebulinic acid on 3T3-L1 adipogenesis at the translational and transcriptional levels. Consistent with published research, adipogenic factors and adipokines were barely expressed before the induction by DMI [39,40,41]. At the translational level, PPARγ and C/EBPα were expressed in the early stage, and their levels increased in the middle stages. Treatment with chebulinic acid decreased the protein expression levels of PPARγ and C/EBPα at every stage and those of adiponectin and aP2 in the middle stages. In the case of translational levels, the results correlated with those of previous studies [19]. In the chebulinic acid-treated group, mRNA expression levels of adipogenic markers, including PPARγ, C/EBPα, FAS, and SCD, were significantly decreased at every stage. In the future, we plan to further identify the anti-obesity effects of chebulinic acid in vivo assays. In addition, the effects of chebulinic acid on other adipogenesis-related pathways, including antagonistic effects on PPARγ, are required to further clarify mechanisms of anti-obesity effects of chebulinic acid. 

In summary, chebulinic acid was selected as a PPP1CB inhibitor and its anti-adipogenic effects were validated. Chebulinic acid suppressed the early stages of adipogenesis, as well as the expression of adipogenic markers at both the protein and mRNA levels, similar to the results observed after the knockdown of PPP1CB. Thus, chebulinic acid is a potent and novel compound for obesity treatment through PPP1CB inhibition.

## 4. Materials and Methods

### 4.1. Purification of PPP1CB

DNA encoding 1–266 residues of human PPP1CB was cloned into the pET28a or pET 30a vector separately. pET28a has an MBP tag and the pET30a vector has a 6xHis or GST tag at the N-terminus. Each vector was transformed into Rosetta (DE3) cells (Merck Millipore, Darmstadt, Germany), and the cells were cultured in Luria–Bertani medium with 1 M sorbitol, 1 mM MnCl_2_, and appropriate antibiotics at 37 °C. After the optical density values reached 0.6, protein expression was induced by adding 1 mM isopropyl-β-D-thiogalactopyranoside with 50 mM betaine and maintained at 12 °C for 3 d. The cells were then harvested and resuspended in buffer A (500 mM NaCl, 50 mM Tris-HCl pH 8.5, phenylmethylsulfonyl fluoride, and 1 mM MnCl_2_) and ultrasonicated to lyse the cells. Subsequently, cell lysates were separated into cell pellets and supernatants, including soluble proteins, by centrifugation at 29,820× *g* at 4 °C for 30 min. For purification of MBP-tagged PPP1CB, the supernatant was added to a column packed with amylose resin (NEB, Essex, MA, USA). After washing the column with buffer A, purified proteins were eluted using elution buffer A (100 mM maltose, 500 mM NaCl, 50 mM Tris-HCl pH 8.5, and 1 mM MnCl_2_). 

To purify GST-tagged PPP1CB, the supernatant was incubated with glutathione resin (Cytiva, Marlborough, MA, USA) in the column. After washing the resins with buffer A, bound proteins were obtained using elution buffer B (10 mM glutathione, 500 mM NaCl, 50 mM Tris-HCl pH 8.5, and 1 mM MnCl_2_). 

To purify 6xHis-tagged PPP1CB, the supernatant was passed through a column packed with talon resin (Takara Korea, Seoul, Korea) five times. After washing the columns with buffer A, elution buffer C (100 mM imidazole, 500 mM NaCl, 50 mM Tris-HCl, pH 8.5, and 1 mM MnCl_2_) was added to the columns to elute the 6xHis-tagged proteins, followed by dialysis with buffer B (50 mM NaCl, 20 mM Tris-HCl, pH 8.5, and 1 mM MnCl_2_) and caspase-3 (1:200 molar ratio to PPP1CB) to cleave the 6xHis tag at 4 °C for 24 h. After dialysis, PPP1CB was separated by Superose-6 column (Cytiva, Marlborough, MA, USA).

### 4.2. Enzymatic Assays 

To estimate the enzymatic activity of PPP1CB, the surrogate substrate DiFMUP, which is widely used to measure the enzymatic activity of protein phosphatases, was used. The concentrations of DiFMUP (800, 400, 200, 100, 50, 25, 12.5, and 6.25 μM) were dissolved in the reaction buffer (20 mM Tris pH 7.0, 150 mM NaCl, 0.01% Triton X-100, and 1 mM MnCl_2_), and protein was added to the reaction buffer containing the indicated concentrations of DiFMUP in a black/black bottom 96-well plate. The fluorescence signals of the wells were monitored for 10 min using a Victor X4 Multilabel Plate Reader (Perkin Elmer, Norwalk, CT, USA) at excitation and emission wavelengths of 355 and 460 nm, respectively. The *K*_M_ value was calculated using the Lineweaver–Burk plots.

To test the inhibitory effects of the 1033 compounds (Chengdu Biopurify, Chengdu, China) on PPP1CB, each compound was dissolved in DMSO (200 μM) and transferred to polypropylene (PP) 96-well plates. Then, 10 μL of each compound was added to 80 µL of reaction buffer containing 304 μM DiFMUP (with 2-fold *K*_M_ values) in a black/black bottom 96-well plate, followed by the addition of 10 μL of 15 nM PPP1CB. The resulting curves were plotted for the product concentration over time.

To determine the IC_50_ values of chebulinic acid for PPP1CB, various final concentrations of chebulinic acid (1.2, 0.6, 0.3, 0.15, 0.75, 0.0038, and 0.0019 μM) were added to the reaction buffer containing DiFMUP (304 μM). Subsequently, 1.5 nM of protein was incubated, and the inhibitor dose–response curves were analyzed using normalized IC_50_ regression curve fitting with control-based normalization.

### 4.3. Cell Culture and Differentiation 

Mouse 3T3-L1 preadipocytes purchased from ZenBio (Durham, NC, USA) were cultured in high-glucose Dulbecco’s modified Eagle’s medium (DMEM; Welgene, Gyeongsan-si, Korea) supplemented with 10% bovine calf serum (Thermo Fisher Scientific Korea Ltd., Seoul, Korea) and antibiotic–antimycotic solution (Welgene, Gyeongsan-si, Korea). Mouse 3T3-L1 preadipocytes were maintained for 24 h after they reached 100% confluency. Differentiation of mouse 3T3-L1 preadipocytes was initiated by culturing in DMEM supplemented with 10% FBS (Welgene, Gyeongsan-si, Korea), antibiotic–antimycotic solution, 0.5 mM IBMX (Merck KGaA, Darmstadt, Germany), 1 µM dexamethasone (Sigma-Aldrich, Saint Louis, MO, USA), and 5 µg/mL insulin (Merck KGaA, Darmstadt, Germany) for 2 days. On day 2, the medium was changed to DMEM supplemented with 10% FBS, antibiotic–antimycotic solution, and 5 µg/mL insulin, and the cells were cultured for another 2 days, followed by maintenance in DMEM supplemented with 10% FBS and antibiotic–antimycotic solution for another 2 days. 

### 4.4. Cell Viability Assays

The cells were cultured in the culture medium until they reached 100% confluency. The cells were then incubated with the indicated concentrations of chebulinic acid (80, 40, 20, or 10 µM) for 2 days. Cell viability assays were conducted using the EZ-CYTOX kit (Daeillab Service, Seoul, Korea) based on WST-1 cell cytotoxicity assays. 

### 4.5. Western Blotting

The expression levels of glucose uptake-related proteins were measured by Western blotting, as described previously [38]. Briefly, 3T3-L1 cells were lysed using radioimmunoprecipitation assay buffer (25 mM 4-(2-hydroxyethyl)-1-piperazineethanesulfonic acid, 150 mM NaCl, 1% Triton X-100, 10% glycerol, 5 mM ethylenediaminetetraacetic acid, 10 mM NaF, 2 mM NaVO_4_, and protease inhibitor cocktail; Roche Korea, Seoul, Korea). Total proteins were resolved using 10% sodium dodecyl sulfate-polyacrylamide gel electrophoresis, transferred to Immobilon-P membranes (Merck KGaA, Darmstadt, Germany) and blotted with the indicated antibodies. The membranes were treated with an anti-rabbit secondary antibody (Cell Signaling Technology, Beverly, MA, USA), followed by detection using enhanced chemiluminescence (ECL) reagents (GE Healthcare Korea, Songdo, Korea) in a WSE-6200 LuminoGraph II (ATTO, Tokyo, Japan). Anti-PPARγ, anti-C/EBPα, anti-adiponectin, and aP2 antibodies were purchased from Cell Signaling Technology (Beverly, MA, USA).

### 4.6. Oil Red O Staining

Oil red O staining of 3T3-L1 adipocytes was performed as previously described, with slight modifications [22]. Briefly, 3T3-L1 pre-adipocytes were seeded into 12-well plates. After they reached 100% confluency, differentiation was induced by media as mentioned above, with 0.1% DMSO as the control and 5, 10, or 20 µM chebulinic acid as the treatment. On day 8, the cells were washed twice with phosphate-buffered saline and fixed with 4% paraformaldehyde for 15 min. After fixation, the cells were stained with 0.3% filtered oil red O solution (Sigma-Aldrich, Saint Louis, MO, USA) for 30 min. The cells were then washed three times with distilled water, and the stained lipid droplets were observed using a Cytation 7 cell imaging multimode reader (Biotek^®^, Winooski, VT, USA). For quantification, oil red O was eluted using 100% isopropanol and the absorbance was measured spectrophotometrically at 520 nm.

### 4.7. RNA Extraction, cDNA Synthesis and RT-PCR

Total RNA was extracted from 3T3-L1 adipocytes using NucleoSpin RNA Plus (Macherey-Nagel, Duren, Germany) following the manufacturer’s protocol. RNA (1 µg) was reverse-transcribed into cDNA using a high-capacity reverse transcription kit (Applied Biosystems, Foster City, CA, USA) according to the manufacturer’s protocol. PCR was performed using SsoAdvanced Universal SYBR Green Supermix (Bio-Rad, Hercules, CA, USA) and a CFX Connect Real-Time PCR Detection System (Bio-Rad, Hercules, CA, USA), according to the manufacturer’s protocol. Primers for RT-PCR were shown in supplementary materials (Table S2).

### 4.8. Statistical Analyses 

Statistical significance (*p* < 0.05) was assessed using a two-tailed unpaired *t*-test for comparisons between two groups and one-way ANOVA for multiple comparisons followed by Tukey’s test. (GraphPad Software, San Diego, CA, USA).

## Figures and Tables

**Table 1:** Kinetic constants depending on the affinity tags.

	[E] (nM)	*K*_M_ (μM)	*V*_max_ (μM min^−1^)	*k*_cat_ (min^−1^)	*k*_cat_*/K*_M_ (μM min^−1^)
GST-1CB ^1^ (A)	5.0	102	3.0	6.0 × 102	5.9
MBP-1CB ^2^ (B)	100	-	0.064	-	-
6xHis-1CB ^3^ (C-1)	10	152.2	3.8	3.8 × 102	2.5
1CB ^4^ (C-3)	1.5	186.2	3.4	2.3 × 103	12.6

^1^ Glutathione-S-transferase-tagged protein phosphatase-1 catalytic subunit beta. The related kinetic data were presented in Figure S4. ^2^ Maltose-binding protein-tagged protein phosphatase-1 catalytic subunit beta. ^3^ 6xHis-tagged protein phosphatase-1 catalytic subunit beta. ^4^ Protein phosphatase-1 catalytic subunit beta without affinity tag. The related kinetic data were presented in Figure S5.

## Data Availability

All the data are provided in the manuscript. Detailed methods and additional data are available upon request from the corresponding authors.

## Supplementary Materials

The following are available online at https://www.mdpi.com/article/10.3390/ijms23020865/s1.
