# Supplementary material for: Chebulinic Acid Suppresses Adipogenesis in 3T3-L1 Preadipocytes by Inhibiting PPP1CB Activity
Source: Int J Mol Sci. 2022 Jan 13;23(2):865. doi: 10.3390/ijms23020865 (PMC8775935; doi:10.3390/ijms23020865)
Supplement: Supplementary file 1 [file ijms-23-00865-s001.zip › ijms-1559419-supplementary.pdf]

Supplementary Information

Chebulinic Acid Suppresses Adipogenesis in 3T3-L1 Preadipocytes by Inhibiting PPP1CB Activity

Jinsoo Kim [1,†], Dohee Ahn [1,†] and Sang J. Chung [1,2,*]

[1] School of Pharmacy, Sungkyunkwan University, Suwon 16419, Korea; neto543@naver.com (J.K.); ehgml94@naver.com (D.A.)
[2] Department of Biopharmaceutical Convergence, Sungkyunkwan University, Suwon 16419, Korea
* Correspondence: sjchung@skku.edu
† These authors contributed equally to this work.

List of Contents

Figure S1. Sequence of purified PPP1CB 2

Figure S2. Purified proteins according to the affinity tags 3

Figure S3. Calibration curve of the fluorescence intensity versus DiFMU 4

Figure S4. Kinetic graph of GST-Tagged-PPP1CB 6

Figure S5. Kinetics graph of PPP1CB without tag 5

Table S1. Information of compounds inhibiting PPP1CB 7

Table S2. Sequences of primers. 8

Figure S6. Certificate of editing. 9

MADGELNVDSLITRLLEVRGCRPGKIVQMTEAEVRGLCIKSREIFLSQPILLELEAPLKICGDIHGQYTDLLRLFEYGGFPPEANYLFLGDYVDR
GKQSLETICLLLAYKIKYPENFFLLRGNHECASINRIYGFYDECKRRFNIKLWKTFTDCFNCLPIAAIVDEKIFCCHGGLSPDLQSMEQIRRIMRPT
DVPDTGLLCDLLWSDPDKDVQGWGENDRGVSFTFGADVVSKFLNRHDLDLICRAHQVVEDGYEFFAKRQLVTLFSAPNYCGEFDNAGGMM
SVDETLMCSFQILKPSEKKAKYQYGGLNSGRPVTPPRTANPPKKR

Figure S1. Sequence of purified PPP1CB (1-266) was highlighted in Red color.

`

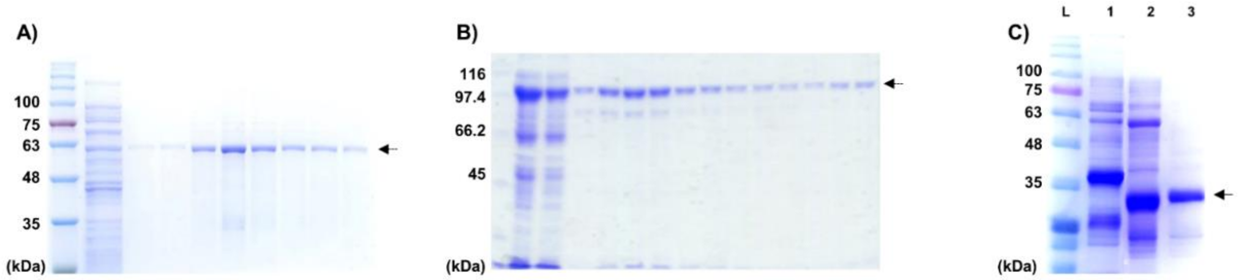

Figure S2. Purified proteins according to the affinity tags. (A) Glutathione-S-transferase (GST)-tagged protein phosphatase-1 catalytic subunit beta (PPP1CB) was purified using the columns packed with glutathione resins. (B) Maltose-binding protein (MBP)-tagged PPP1CB was purified using amylose resins. (C) 1 - 6xHis-tagged PPP1CB was purified using talon resins. 2 - 6xHis tag was cleaved using caspase-3. 6xHis tag and caspase-3 were eliminated using talon resins. 3 - PPP1CB without any affinity tag was subjected to further purification using Superose-6.

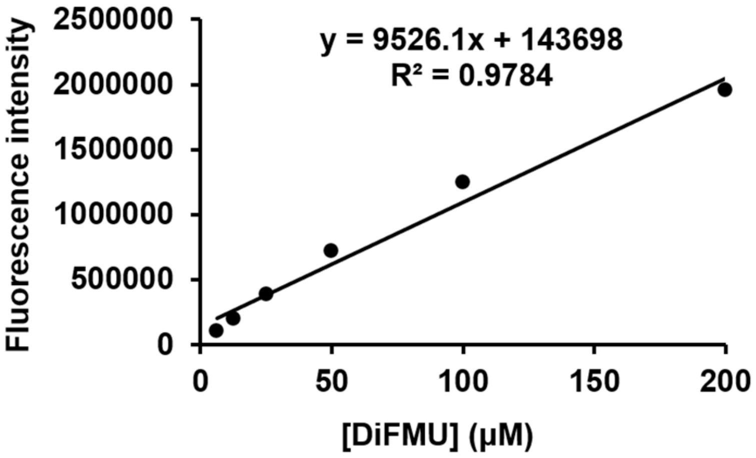

Figure S3. Calibration curve of the fluorescence intensity versus DiFMU from 6.25 to 200 μM. DiFMU with various concentrations (6.25, 12.5, 25, 50, 100, 200 μM) in the reaction buffer (20 mM Tris pH 7.0, 150 mM NaCl, 0.01% Triton X-100, and 1 mM MnCl2) was detected by a Victor X4 Multilabel Plate Reader at excitation and emission wavelengths of 355 and 460 nm.

`

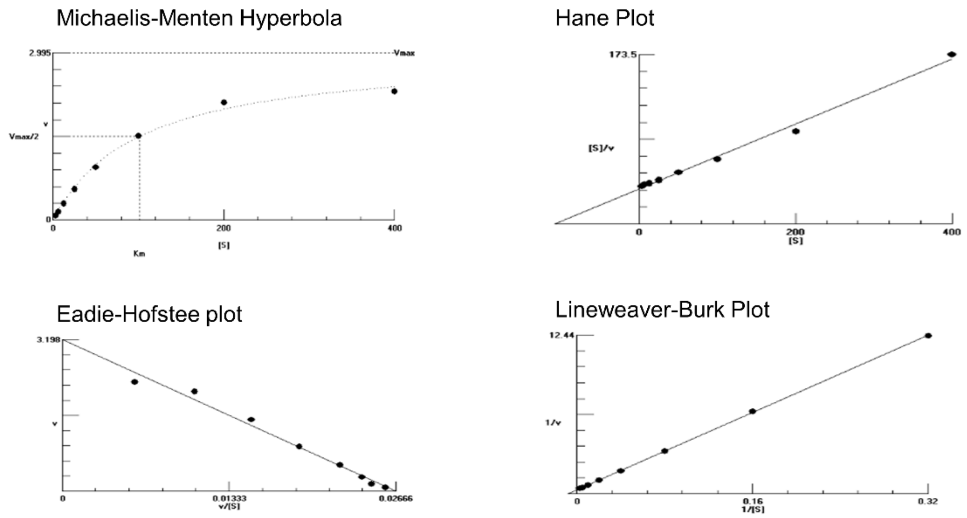

Figure S4. Kinetic graph of GST-Tagged-PPP1CB. Protein was added to reaction buffer containing each concentration of DifMUP (400, 200, 100, 50, 25, 12.5, 6.25, and 3.13 μM).

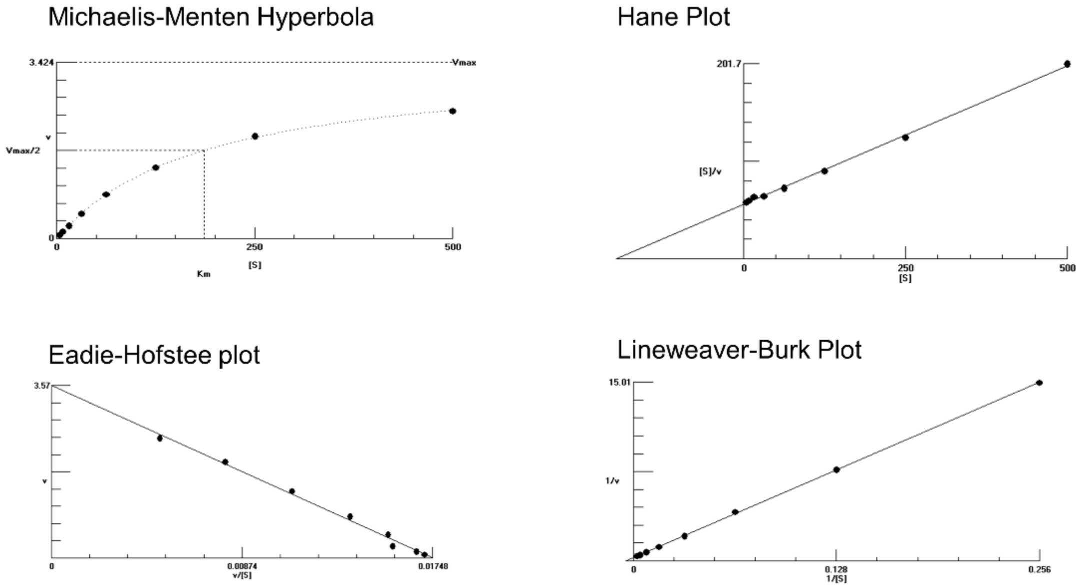

Figure S5. Kinetics graph of PPP1CB without tag. Protein was added to reaction buffer containing each concentration of DifMUP (500, 250, 125, 62.5, 31.25, 15.63, 7.81, and 3.91 μM).

Table S1. Information of compounds inhibiting PPP1CB.

Compound number	Compound name	CAS number
1	1,2,3,4,6-o-Pentagalloylglucose	30964-13-7
53	Acetylshikonin	24502-78-1
65	Alpha-Boswellic acid	471-66-9
69	Amentoflavone	1617-53-4
100	Baicalein	491-67-8
132	Cantharidin	56-25-7
142	Celastrol	34157-83-0
146	Chebulagic acid	23094-71-5
147	Chebulinic acid	18942-26-2
164	Coptisine Chloride	6020-18-4
176	Crocetin	27876-94-4
177	Crocin I	42553-65-1
178	Crocin II	55750-84-0
189	Cyanidin chloride	528-58-5
190	Cyanidin-3-glucoside chloride	7084-24-4
235	Elllagic Acid	476-66-4
242	Epigallocatechin gallate	989-51-5
261	Fisetin	528-48-3
271	Gallic Acid Ethyl Ester	831-61-8
272	Gallocatechin gallate	4233-96-9
284	Geraniin	60976-49-0
331	Hypericin	548-04-9
335	Icariside I	56725-99-6
340	Irinotecan Hydrochloride	100286-90-6
362	Juglone	481-39-0
410	Medicagenic acid	599-07-5
428	Myricetin	529-44-2
429	Myricetrin	17912-87-7
471	Palmatine Chloride	10605-02-4
526	Punicalagin	65995-63-3
527	Punicalin	65995-64-4
528	Purpurin	81-54-9
531	Quercetin 7-rhamnoside	22007-72-3
558	Salvianolic acid C	115841-09-3
574	Scopoletin	92-61-5
576	Scutellarein	529-53-3
582	Sennoside A	81-27-6
583	Sennoside B	128-57-4
670	Ligustilide	81944-09-4
721	Sulforaphene	592-95-0
750	Dimethoxylapigenin	5128-44-9
991	Epiberberine	6873-09-2
1044	Luteolin	491-70-3
1053	Norcantharidin	29745-04-8
1088	Soyasaponin Ba	114590-20-4

Table S2. Sequences of primers.

Gene	Forward (5'-3')	Reverse (5'-3')
Mouse *PPARγ*	AGGGCGATCTTGACAGGAAA	GATCGAAACTGGCACCCTTG
Mouse *C/EBPα*	GTGGACAAGAACAGCAACGAG	TTGACCAAGGAGCTCTCAGG
Mouse *FAS*	GCTGCGGAAACTTCAGGAAA	GAGTTGAGCTGGGTTAGGGT
Mouse *SCD*	CGAGAGAAGGTGAAGACGGT	TAGAGCTTGCAGGAGGGAAC

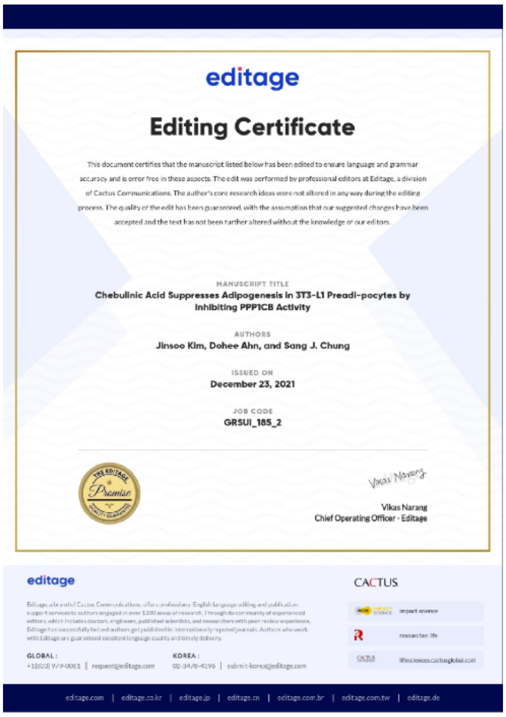

editage

Editing Certificate

This document certifies that the manuscript listed below has been edited to ensure language and grammar accuracy and is error free in these aspects. The edit was performed by professional editors at Editage, a division of Cactus Communications. The author's core research ideas were not altered in any way during the editing process. The quality of the edit has been guaranteed, with the assumption that our suggested changes have been accepted and the text has not been further altered without the knowledge of our editors.

MANUSCRIPT TITLE
Chebulinic Acid Suppresses Adipogenesis in 3T3-L1 Preadi-pocytes by Inhibiting PPP1CB Activity

AUTHORS
Jinsoo Kim, Dohee Ahn, and Sang J. Chung

ISSUED ON
December 23, 2021

JOB CODE
GRSUI_185_2

THE EDITAGE Promise QUALITY GUARANTEE

Vikas Narang
Chief Operating Officer - Editage

editage

Editage, a brand of Cactus Communications, offers professional English language editing and publication support services to authors engaged in over 1300 areas of research. Through its community of experienced editors, which includes doctors, engineers, published scientists, and researchers with peer review experience, Editage has successfully helped authors get published in internationally reputed journals. Authors who work with Editage are guaranteed excellent language quality and timely delivery.

GLOBAL : +1(833) 979-0061 | request@editage.com
KOREA : 02-3478-4396 | submit-korea@editage.com

CACTUS
impact.science
researcher.life
lifesciences.cactusglobal.com

editage.com | editage.co.kr | editage.jp | editage.cn | editage.com.br | editage.com.tw | editage.de

Figure S6. Certificate of editing.
